# Supplementary material for: Isolation and Enumeration of CTC in Colorectal Cancer Patients: Introduction of a Novel Cell Imaging Approach and Comparison to Cellular and Molecular Detection Techniques
Source: Cancers (Basel). 2020 Sep 16;12(9):2643. doi: 10.3390/cancers12092643 (PMC7563529; doi:10.3390/cancers12092643)
Supplement: Supplementary file 1 [file cancers-12-02643-s001.pdf]

Supplementary Methods

Detection of CTC by the NYONE® cell imager:

For the purpose of establishing a semi-automated microscopic detection by the cell imager NYONE® (SYNENTEC, Elmshorn, Germany), HT29 cancer cells were kept in culture according to the general standard with Roswell Park Memorial Institute (RPMI)-1640 Medium (Gibco, Thermo Fisher Scientific, Darmstadt, Germany), 10% fetal calve serum (FCS) (Merck, Darmstadt, Germany), 1 mM sodium pyruvate (Gibco) and 2 mM glutaMAX (1 fold +L-Glutamin + 25 mM HEPES + 10 % FCS) (Gibco) at 5% CO_2 and 37°C. They were trypsinised and fixed using fixation buffer (#14190-094, Biolegend, San Diego, CA, USA) diluted 1:4 in PBS/1% FCS. After cell counting using NYONE®, an average of 100 HT29 cells (determined by repeated counting) were then spiked into 8.2 ml of healthy donors´ blood samples and then transferred to BD Vacutainer® Mononuclear Cell Preparation Tubes (CPT) (Becton Dickinson, Heidelberg, Germany). For isolation of the mononuclear cell (MNC) fraction, the CPT tubes were centrifuged at 1650xg for 20 minutes. After transferal of the MNC to 50 ml centrifuge tubes, a fixation buffer (#14190-094, Biolegend, San Diego, CA, USA) was added and incubated for 15 minutes at room temperature after which the samples were stored at 4°C for up to four days until further analysis was possible.

For final quantification of CTC, at first, the cells were permeabilised with a Perm-/Wash-Buffer (#421002, Biolegend) for 5 minutes at room temperature and then centrifuged at 330xg for 10 minutes. Then, the cells were incubated with an Fc-blocking buffer (#422301/2, Biolegend) for 15 minutes. Hereafter, the cells were incubated for 30 minutes with the antibodies: anti-CD45-AF488 (#304017; Biolegend), anti-EpCAM-AF647 (#324212; Biolegend), anti-pan-Cytokeratin-AF647 (#628604; Biolegend), anti-EGFR-AF647 (#sc-120 AF647; SantaCruz, Dallas, TX, USA), anti-Her2-AF647 (#3244412; Biolegend). Finally, a buffer containing DAPI (1:10,000) (#422801; Biolegend) was added to the cells.

Now, the suspension containing the cells was successively transferred to a 96-well plate (Sarstedt, Darmstadt, Germany), so that each well was filled with 200µl of sample solution. The 96-well plates were then centrifuged at 330xg for 10 minutes and afterwards placed in the NYONE® cell imager for analysis (**Figure 1A**).

At first, the plates were scanned only for the detection of Alexa647 fluorescence (Ex 632/22, Em 685/40) as only CTCs should be positive in this setting. SYNENTEC's proprietary YT®-Software (SYNENTEC) automatically analysed the images already during scanning and detected positive events. The image processing settings for this analysis were determined using the samples with spiked tumour cells. Each event was then automatically further analysed in depth by creating a region of interest (ROI) around it. This ROI was scanned in four channels (DAPI: Ex 377/50 Em 452/45, Alexa488: Ex 475/28 Em 530/43,

Alexa-647: Ex 632/22 Em 685/40, brightfield: Ex brightfield Em blue). YT®-Software then automatically detected the cells´ nuclei (DAPI, blue fluorescence) and analysed whether a virtual cytoplasm surrounding the nuclei was fluorescing green (CD45) or red (EpCAM, Pan-CK, EGFR, Her2). These events were finally presented separately by the software and the investigator was able to examine the morphology of the potential CTC (**Figure 1A and 2A**).

Patients´ blood samples were handled equally and as described above.

Immunofluorescence assays used to immunostain CTC on the porous membrane (ScreenCell®):

The colon cancer cell line HT29 was used to immunostain a panel of cytokeratins (pan-CK, Clone: AE1/AE3, #M3515; Agilent, Santa Clara, CA, USA). The HT29 cells were enriched and fixed with 2% formalin on a porous membrane named Cyto isolation support or Cyto IS using ScreenCell® Cyto kit (ScreenCell, Sarcelles, France) from 3 ml of blood of healthy donors (**Figure 1B and 2B**). The Cyto IS device was dried during one hour at 37°C and stored at 4°C. Prior to the immunofluorescence staining, the Cyto IS device with HT29 cells was hydrated with Tris-Buffered Saline (TBS) (#S30001; Agilent). The antigens were retrieved with target retrieval solution pH 6 (#S1700; Agilent) at 95-99˚C for 20 min, cooled during 20 minutes at room temperature and washed with TBS. The isolated cells were treated with a blocking solution containing 3% BSA in TBS during 30 minutes at room temperature. Then, the ScreenCell® Cyto IS device was incubated overnight at 4°C with anti-pan-CK mouse monoclonal antibody (#M3515; Agilent) and anti-CD45 (Clone EP68) rabbit monoclonal antibody (#AC-0065A; Epitomics, Abcam, Cambridge, GB). The ScreenCell® Cyto IS device was washed twice with TBST (#S3306, Agilent) and incubated for one hour at room temperature with secondary antibodies goat anti-mouse IgG Alexa Fluor488 (#A11001; Life Technologies, Carlsbad, CA, USA) and goat anti-rabbit IgG Alexa Fluor568 (Cat# A11011; Life Technologies). The ScreenCell® Cyto IS was then washed twice and incubated with DAPI (#D1306, Life Technologies) during 10 minutes to counterstain the nucleus. After a final wash with TBST the ScreenCell® Cyto IS device was mounted with the Vectashield® (#H-1000; Vector Laboratories, Burlingame, CA, USA). The microscopic analysis was performed with Epi-fluorescent microscope (Nikon Eclipse E80i, Tokyo, Japan) equipped with NIS-Elements Viewer software (Nikon). Mx obecive (obj.) 40.

Blood samples from CRC patients were processed using ScreenCell® Cyto kit and were firstly stained with May Grunwald Giemsa (MGG) (RAL Diagnostics, Martillac, France). The CTC were analysed after microscopic scanning and counted.

The cells were than incubated in TBST to remove MGG and cells were immunostained according to the immunofluorescence protocol applied on spiked HT29 cells. The isolated atypical cells that were pan-CK-negative/CD45-negative, pan-CK-positive/CD45-negative or pan-CK-positive/CD45-positive, were

enumerated by an experienced pathologist with Epi-fluorescent microscope (Nikon Eclipse E80i) equipped with NIS-Elements Viewer Software. Mx obj. 40.
